# The development of a standard training toolkit for research studies that recruit pregnant women in labour

**DOI:** 10.1186/1745-6215-14-362

**Published:** 2013-10-30

**Authors:** Sara Kenyon, Jackie Sears, Hannah Reay

**Affiliations:** 1 School of Health and Population Sciences, University of Birmingham, Birmingham B15 2TT, UK; 2 National Institute for Health Research Pan Birmingham Cancer Research Network, Old Queen Elizabeth Hospital, Mindelsohn Way, Birmingham B15 2TH, UK; 3 National Institute for Health Research Birmingham and the Black Country Comprehensive Local Research Network, Institute of Research and Development, Birmingham Research Park, Birmingham B15 2SQ, UK

**Keywords:** Research training, Regulatory requirements

## Abstract

Recruitment of pregnant women in labour to clinical trials poses particular challenges. Interpretation of regulation lacks consistency or clarity and variation occurs as to the training required by clinicians to safely contribute to the conduct of intrapartum studies. The Royal College of Obstetricians and Gynaecologists Intrapartum Clinical Study Group initiated the development of a pragmatic, proportionate and standardised toolkit for training clinical staff that complies with both regulatory and clinician requirements and has been peer-reviewed. This approach may be useful to researchers in acute care settings that necessitate the integration of research, routine clinical practice and compliance with regulation.

## Correspondence/Findings

### Letters

#### Background

Over-regulation of clinical trials has been well documented as hindering the delivery of research [1,2] and extends to the training required by clinical (non-research) staff undertaking consent and recruitment processes during their routine practice. This can present particular challenges as uncertainty may exist for researchers regarding what training is necessary to uphold the principles of Good Clinical Practice (GCP), what is practical in the context of clinical workload, and what is deemed to be ‘sufficient’ training by sponsors, host organisations and regulators.

Variations in training requirements for clinicians were identified as part of the High or Low Dose Syntocinon (HOLDS) pilot study [3]. This was a pilot for a trial where eligible women were in delayed labour and could be identified at any time of day; recruitment needed to occur within a short window period, so to maximize recruitment clinicians needed to recruit women at any time. While standard study-specific training was developed by the Trial Management Group for use at the collaborating sites, whether clinicians were permitted to recruit women in labour over the 24-hour period varied; this was decided by the local Research and Development Departments and hinged on the amount of training they felt was required to undertake predefined research tasks, such as taking consent in labour. Some adopted a pragmatic approach whereby clinically experienced staff who had received study-specific training (including tailored Good Clinical Practice (GCP) and consent training), could recruit women over the 24-hour period. Others stipulated that only research-experienced staff were able to recruit and that to be deemed such, they must have undertaken an approved GCP course, which is usually a day in length. At these sites it was not feasible for all clinicians on Delivery Suite (usually numbering in excess of 100 clinical staff) to receive such training; recruitment was therefore restricted to office hours, which not only limited the numbers of women recruited, but also meant that for clinical staff there was a clear division between research and practice.

Whilst United Kingdom (UK) legislation indicates that everyone involved in conducting a trial should be qualified by education, training and experience to perform their respective tasks [4], no guidance on the content of training could be found that met both regulatory requirements and clinical needs. Discussions within the Royal College of Obstetricians and Gynaecologists (RCOG) Intrapartum Clinical Study Group identified the need to define suitable training that should enable clinicians to safely contribute to the conduct of intrapartum research studies. This led to the development of a training toolkit in collaboration with the National Institute for Health Research (NIHR) Clinical Research Network GCP facilitators. This training toolkit is available at http://www.bmfms.org.uk/News/Intrapartum-CSG-develop-research-toolkit-/c-1-1-123.

#### Contents of the toolkit

The materials within the toolkit promote an awareness of GCP, in particular, consent and safety procedures, whilst acknowledging that training should be proportionate and tailored to the role of the clinician in the study. It does not replace the need for more detailed GCP training as applicable to Principal Investigators or other research staff who will provide local research expertise within a site.

The toolkit highlights the need for chief investigators (CIs) and principal investigators (PIs) to consider the duties and tasks that may be undertaken by clinical staff during routine intrapartum care (for example, checking eligibility, receiving consent as part of an ongoing process, study specific interventions or data collection).The toolkit provides appropriate study specific training in accordance with the principles of GCP. Standard training materials are approved by the sponsor, in consultation with collaborators, and would not be altered by sites.

The toolkit includes: a PowerPoint presentation template (28 slides into which study-specific information is added, and which includes quick questions to check understanding and encourage debate); Guidance Notes to support the use of the presentation template; and a Template Training File (word document template for a variety of study-specific reference materials).

The content of the GCP training slides informs clinicians that these are the fundamental standards that underpin clinical research to ensure the safety of research participants and the integrity of the data. These standards stress that the study must be conducted in compliance with the protocol that has received regulatory and local hospital approval. The training reminds clinicians of their responsibility to ensure the safety and wellbeing of research participants, and that, as in clinical practice, the clinicians should not undertake any tasks for which they have not been trained and are unwilling to undertake.

Consent is endorsed as being a continuous process. The slides outline the principles of consent in research and the documentation of this process. They further detail the advice from the RCOG regarding the timing of information for women depending on the frequency of the condition being researched [5]. The toolkit reiterates that those receiving consent should do so in their normal practice, be familiar with the study, be able to answer any questions the women may have and sign to confirm they have received training. The training toolkit also includes the importance of documenting the consent process and of safety reporting; study-specific detail can be added to the slide templates.

## Conclusions

This initiative has resulted in a pragmatic, proportionate and standardised approach to study-specific training which fulfills both regulatory requirements and the needs of clinical staff and facilitates recruitment. It enables intrapartum research to be embedded in clinical practice and provides a useful template for researchers in other acute clinical settings such as critical care.

## Abbreviations

CI: Chief investigator; GCP: Good clinical practice; HOLDS: High or low dose syntocinon pilot study; NIHR: National Institute for Health Research; PI: Principal investigator; RCOG: Royal College of Obstetricians and Gynaecologists.

## Competing interests

SK is the Chair of the Royal College of Obstetricians and Gynaecologists Intrapartum Clinical Study Group, and she initiated and led the development. HR and JS are NIHR GCP Facilitators.

## Authors’ contributions

SK led the development with HR and JS. SK drafted the manuscript, which was commented on by HR and JS. All authors read and approved the final manuscript.

## Authors’ information

SK is funded by the National Institute for Health Research (NIHR) Collaboration for Leadership in Applied Health Research and Care in Birmingham and the Black Country.

This article/paper/report presents independent research funded by the National Institute for Health Research (NIHR). The views expressed are those of the author(s) and not necessarily those of the NHS, the NIHR or the Department of Health.
